# slag: A program for seeded local assembly of genes in complex genomes

**DOI:** 10.1111/1755-0998.13580

**Published:** 2022-01-27

**Authors:** Charles F. Crane, Jill A. Nemacheck, Subhashree Subramanyam, Christie E. Williams, Stephen B. Goodwin

**Affiliations:** ^1^ USDA‐Agricultural Research Service Crop Production & Pest Control Research Unit Purdue University campus West Lafayette Indiana USA; ^2^ 311308 Department of Botany & Plant Pathology Purdue University West Lafayette Indiana USA; ^3^ 311308 Department of Entomology Purdue University West Lafayette Indiana USA

**Keywords:** bioinfomatics/phyloinfomatics, long reads, multiple alleles, pipeline, sequence assembly

## Abstract

Although finished genomes have become more common, there is still a need for assemblies of individual genes or chromosomal regions when only unassembled reads are available. slag (Seeded Local Assembly of Genes) fulfils this need by performing iterative local assembly based on cycles of matching‐read retrieval with blast and assembly with cap3, phrap, spades, canu or unicycler. The target sequence can be nucleotide or protein. Read fragmentation allows slag to use phrap or cap3 to assemble long reads at lower coverage (e.g., 5×) than is possible with canu or unicycler. In simple, nonrepetitive genomes, a slag assembly can cover a whole chromosome, but in complex genomes the growth of target‐matching contigs is limited as additional reads are consumed by consensus contigs consisting of repetitive elements. Apart from genomic complexity, contig length and correctness depend on read length and accuracy. With pyrosequencing or Illumina reads, slag‐assembled contigs are accurate enough to allow design of PCR primers, while contigs assembled from Oxford Nanopore or pre‐HiFi Pacific Biosciences long reads are generally only accurate enough to design baiting sequences for further targeted sequencing. In an application with real reads, slag successfully extended sequences for four wheat genes, which were verified by cloning and Sanger sequencing of overlapping amplicons. slag is a robust alternative to atram 2 for local assemblies, especially for read sets with less than 20× coverage. slag is freely available at https://github.com/cfcrane/SLAG.

## 1 INTRODUCTION

There are three major options for investigating a gene or gene family when no good genome assembly exists but reads are available. One option is to wait for a finished genome to be released; another option is to parse raw reads that match a homeologous gene sequence from a species with a better assembly. In between there is a third option, to assemble contigs from a set of reads that match the sequence of the target gene. An extension of this third option is an iterative local assembly based on cycles of matching‐read retrieval and assembly.

Several programs have been developed to perform iterative local assembly, starting with tracembler (Dong et al., 2007), which retrieves reads from the NCBI Trace Archive database and then uses the retrieved reads in subsequent queries against the same database, finally assembling contigs from the accumulated reads with cap3 (Huang & Madan, 1999). beap (Koltes et al., 2009) follows the same strategy with additional NCBI databases and can also retrieve reads from a local database. The program tasr (Targeted Assembly of Sequence Reads), while not itself iterative, nevertheless illustrates another strategy for local assembly in which reads are selected if their first 15 bases exactly match any 15‐base subsequence in the targeted sequences (Warren & Holt, 2011). The reads are then aligned and a consensus sequence is built up by majority vote at each nucleotide position. mapsembler (Peterlongo & Chikhi, 2012) is similar but uses k‐mer words anywhere in the reads and iteratively collects sets of reads for attaching short matching subsequences to both ends of the growing, resulting contig. Another in silico strategy for reconstructing complete mitochondrial genomes from NGS data is mitobim (mitochondrial baiting and iterative mapping; Hahn et al., 2013). Reads matching 31‐nucleotide starting sequences are retrieved and assembled initially into contigs with mira (Chevreux et al., 1999), and subsequent iterations find and assemble reads that overlap the ends of the growing contigs. grabb (Brankovics et al., 2016) also retrieves reads on the basis of matching 31‐mer words and then assembles all the retrieved reads with edena (Hernandez et al., 2008) or velvet (Zerbino & Birney, 2008). grabb repeats this cycle until contigs completely match the seeding sequence(s) or until no additional reads are retrievable. kollector (Kucuk et al., 2017) uses 48‐base k‐mers from seeding sequences to populate a Bloom filter that selects matching reads, from which additional k‐mers are selected to update the Bloom filter for the next iteration of matching; the final read collection is assembled with abyss (Simpson et al., 2009). The programs tram (Johnson et al., 2013), atram (Allen et al., 2015) and atram2 (Allen et al., 2018) implement the full cycle of read retrieval with blast (Zhang et al., 2000), assembly with abyss (Simpson et al., 2009), velvet (Zerbino & Birney, 2008), trinity (Grabherr et al., 2011) or spades (Bankevich et al., 2012), and seeding of the next cycle with contigs that match the target sequence. srassembler (McCarthy et al., 2019) follows a cycle of read retrieval with vmatch (Abouelhoda et al., 2004), contig assembly with abyss (Simpson et al., 2009) or soapdenovo2 (Luo et al., 2012), selection of contigs to retrieve the next set of reads, and periodic purging of contigs that do not match the target sequence. srassembler can also defer the first assembly until multiple cycles of read retrieval have been completed, using previously retrieved reads as query sequences to retrieve more reads.

Although many local assemblers are available, none deals explicitly with: (i) long reads, as are now available from Pacific Biosciences or Oxford Nanopore sequencing; (ii) hybrid assemblies containing both long and short reads; and (iii) sequencing projects with shallow read depth. Therefore, we introduce slag (Seeded Local Assembly of Genomes), a versatile, command‐line, local assembly pipeline that can assemble reads of any length and coax assemblies from shallow read sets. slag is similar to atram (Allen et al., 2015) and atram2 (Allen et al., 2018) in that it uses blastn or blastx to identify matching reads, then assembles them with phrap (Green, P., http://www.phrap.org/phredphrap/phrap.html), cap3 (Huang & Madan, 1999), spades (Bankevich et al., 2012), canu (Koren et al., 2017) or unicycler (Wick et al., 2017), and carries forward to the next cycle the contigs that match the target sequence by blastn or tblastn search. However, slag has its own nuances to increase contig length and can utilize a fragmentation strategy with phrap or cap3 to assemble long reads at low (e.g., 5×) coverage, where canu and unicycler fail to produce an assembly.

## 2 METHODS

### 2.1 Work flow

#### 2.1.1 Input files

slag is written in Perl 5.16. slag reads all necessary settings and file names from a user‐supplied configuration file, which must also be written in syntactically correct Perl. Thus the user has control over the stringency of read retrieval and assembly. A blastable database of reads is always needed and must be supplied by the user. Depending on the chosen assembler, a fastq file of paired‐end reads might also be needed. If contigs are to be polished (error‐corrected with more accurate short reads), a combined file of fastq reads is needed, with all reads having distinct names. slag also requires a query file of one or more nucleotide or protein sequences in fasta format. Since all retrieved reads are assembled together, it is probably more efficient to execute separate runs for individual or small groups of query sequences than to run thousands at once, but slag itself imposes no limit on the number of query sequences. Depending again upon the chosen assembler, it might be necessary to set up the environment with an appropriate loading command before running slag.

#### 2.1.2 Read gathering

slag identifies target‐matching reads in the blastable database with blastn or tblastn, then retrieves them with blastdbcmd. While the user specifies the e‐value and maximum number of reads to retrieve, the number typically far exceeds the number of reads actually assembled. slag limits the number of reads assembled in any one of five ways: (i) all, where all retrieved reads are used; (ii) bitscore, where only reads that match higher than a specified blast bitscore are used; (iii) increment, where the count of reads used increases by a constant value (the increment) with each cycle; (iv) population, where reads are accepted at consecutively decreasing bitscores until all reads from the previous cycle have been included; and (v) manual, where the user directly specifies the number of reads to be assembled at each cycle. With the increment and manual options, the reads are taken from the top of a list in decreasing order of blast bitscore.

#### 2.1.3 Assembly

slag performs a de novo assembly of all selected reads at each cycle. Thus there is continuous interaction among nascent contigs and the read set that determines which reads contribute to which contigs. slag can use spades (Bankevich et al., 2012) for short reads, phrap (Green, 1999) or cap3 (Huang & Madan, 1999) for short or medium‐length reads, and canu (Koren et al., 2017) or unicycler (Wick et al., 2017) for long reads. slag can also fragment long reads in two ways for assembly with phrap or cap3 and thereby bypass the minimum read depth required for canu or unicycler. In the first, reads are broken without overlap into pieces of a user‐specified length (e.g., 600 bases) and then assembled. In the second way, reads are broken without overlap into pieces of one length (e.g., 610 bases), and the intact reads are also broken into pieces of a second length (e.g., 490 bases). All of the pieces are then assembled together. This doubles the apparent read depth for the assembler without adding any new information. It merely allows the assembly to span regions of 1–2× coverage that would otherwise interrupt contig growth.

#### 2.1.4 Contig selection and polishing

slag produces a blastable database from the contigs and queries the original seeding sequences against it with blastn or tblastn. Only the matching contigs pass on to the next cycle. slag can polish seeding contigs with racon (Vaser et al., 2017) for a user‐specified number of iterations. In this case, the polished contigs proceed to the next cycle. Since racon requires a .sam file of read alignments to the contigs, slag uses bowtie2‐build and bowtie2 (Langmead & Salzberg, 2012) to produce the .sam file.

#### 2.1.5 Stopping criteria

The user can specify how slag stops: (i) after a set number of cycles; (ii) when the longest contig fails to lengthen by a minimum amount from the previous cycle; or (iii) if all manually set read counts have been used. slag will also stop if no contigs are generated or if no generated contig sufficiently matches the target sequence.

#### 2.1.6 Output files

For each cycle of retrieval and assembly, slag outputs a fasta file of the assembled contigs that match the target seed sequence. If contigs have been polished with racon, the polished contigs will appear in a separate file. slag also outputs a log file listing all calls to blast, the assembler and the polisher.

### 2.2 Simulations

Contig length and accuracy were tested in three sets of simulations. The first set simulated very similar variants of a 7‐kb random founding sequence generated with nucleotide frequencies drawn from a Hessian fly‐responsive, dirigent‐like sequence (GenBank accession JX501668.1, Subramanyam et al., 2013) from bread wheat (*Triticum aestivum* L.). The sequence consisted of a relatively conserved central region of 1800 nucleotides flanked by less conserved sequence; individual variants were derived from the founding sequence by random mutations with respective probabilities of 0.005 and 0.02 per nucleotide in the central and flanking regions. Simulated read lengths were distributed as in the pyrosequencing (454) reads used by Brenchley et al. (2012) to assemble the genome of Chinese Spring wheat. Read depth and accuracy were varied to determine the effect on contigs generated with phrap (Green, 1999).

The second set drew simulated reads of various lengths at random from three homeologous regions of chromosomes 1A, 1B and 1D of version 2.0 of the genome of hexaploid bread wheat (*T*. *aestivum* L.) as downloaded from https://urgi.versailles.inra.fr/download/iwgsc/IWGSC_RefSeq_Assemblies/v2.0/. This set allowed investigation of the response of contig length to abundant repetitive elements. The regions consisted of nucleotides 559066659–596637530 in chromosome 1A, 627849254–696738616 in chromosome 1B, and 454010413–496939616 in chromosome 1D. These homeologous regions sum to 149,389,439 nucleotides. While eight of the 50 seed sequences were oriented more than one way, none of them had the same relationship of orientation to chromosome as any of the others, so there was no convincing evidence for large‐scale chromosomal inversions among the three sampled regions. Distinct perl scripts were written to generate long (7–14 kb) single reads and short (150 bp) paired‐end reads. Random variations were introduced to simulate sequencing errors, including substitutions, single‐base deletions, single‐base insertions, and shifted count in runs of a single nucleotide. For paired‐end reads, the respective probabilities of these errors per base were 0.005, 0.001, 0.001 and 0.01, leading to an overall error probability somewhat less than 0.01 per base. For long reads, these respective probabilities were 0.07, 0.01, 0.01 and 0.80, resulting in an overall match of about 90% by blastn alignment or an overall error probability of about 0.10. These error frequencies were intended respectively to simulate reads generated by Illumina and OxfordNanopore sequencing technologies. For choosing the 50 seed sequences, the coordinates of gene models were downloaded as a gff file for version 1.0 of the wheat genome (International Wheat Genome Sequencing Consortium, 2018) from https://urgi.versailles.inra.fr/download/iwgsc/IWGSC_RefSeq_Annotations/v1.0/. Sequence for Chinese Spring version 1.0 was downloaded from https://urgi.versailles.inra.fr/download/iwgsc/IWGSC_RefSeq_Assemblies/v1.0/. A perl script was written to extract sequence for 50 version 1.0 gene models in the version 1.0 sequence. Each of these gene models exists at least once in the sampled sequence from version 2.0. The closest blast hits for these wheat sequences in the Genbank nr database are given in Table 1.

**TABLE 1:** Wheat gene models used as target sequences for local assembly

Gene Mmodel	e‐ value	Accession no.	Description
TraesCS1A01G393300.1	6e‐21	EMS62516.1	Hypothetical protein TRIUR3_20468
TraesCS1A01G393800.1	6e‐29	EMS54359.1	Chaperone protein DnaJ
TraesCS1A01G396600.1	9e‐24	XP_020158285.1	E3 SUMO‐protein ligase MMS21
TraesCS1A01G396600.2	2e‐21	XP_020158285.1	E3 SUMO‐protein ligase MMS21
TraesCS1A01G397600.1	7e‐55	XP_020153608.1	Zinc finger MYM‐type protein 1‐like
TraesCS1A01G398400.1	8e‐44	XP_020169217.1	ras‐related protein RABA1f‐like
TraesCS1A01G399600.1	No hit		
TraesCS1A01G402200.2	4e‐29	EMS60683.1	General transcription factor 3C polypeptide 2
TraesCS1A01G403200.1	2e‐28	XP_020147766.1	FRIGIDA‐like protein 3
TraesCS1A01G404500.1	9e‐26	XP_020176384.1	Phytepsin
TraesCS1A01G405600.1	6e‐45	XP_020176342.1	Sugar transporter ERD6‐like 4
TraesCS1A01G407000.1	8e‐44	VAH10327.1	Unnamed protein product
TraesCS1A01G408800.1	2e‐44	XP_020186556.1	Short‐chain dehydrogenase/reductase 2b‐like
TraesCS1A01G410200.1	No hit		
TraesCS1A01G411500.2	2e‐36	VAH11092.1	Unnamed protein product
TraesCS1A01G411700.1	3e‐33	XP_020174231.1	Nuclear transcription factor Y subunit B‐4‐like
TraesCS1A01G415200.1	1e‐32	KAE8800973.1	Protein CHUP1, chloroplastic
TraesCS1A01G417300.1	2e‐43	XP_020154488.1	Peroxisomal membrane protein PEX14‐like isoform X2
TraesCS1A01G419700.1	6e‐27	XP_020178694.1	Uncharacterized protein LOC109764261 isoform X2
TraesCS1A01G421800.1	No hit		
TraesCS1A01G423100.1	1e‐25	EMS51643.1	Spastin
TraesCS1A01G423800.1	1e‐59	EMS58218.1	Late embryogenesis abundant protein Lea14‐A
TraesCS1A01G426400.1	5e‐39	VAH11437.1	Unnamed protein product
TraesCS1A01G430700.1	No hit		
TraesCS1A01G430700.3	6e‐43	XP_020175200.1	Trimethylguanosine synthase‐like isoform X2
TraesCS1A01G431300.1	2e‐38	XP_020187356.1	Proteinase inhibitor PSI‐1.2‐like
TraesCS1A01G433600.1	3e‐50	KAE8773310.1	Disease resistance protein RGA2
TraesCS1A01G437500.2	No hit		
TraesCS1A01G439200.1	1e‐26	VAH11693.1	Unnamed protein product
TraesCS1A01G441500.1	2e‐42	VAH11737.1	Unnamed protein product
TraesCS1A01G442400.2	8e‐51	XP_020190989.1	TATA‐binding protein‐associated factor BTAF1‐like
TraesCS1A01G444100.1	4e‐40	XP_020157674.1	Two‐component response regulator ORR42‐like
TraesCS1B01G397300.1	9e‐50	VAH22346.1	Unnamed protein product
TraesCS1B01G400200.1	3e‐30	XP_020197381.1	65‐kDa microtubule‐associated protein 3‐like
TraesCS1B01G401300.1	1e‐49	XP_020187904.1	Oligopeptide transporter 7‐like isoform X4
TraesCS1B01G402400.1	No hit		
TraesCS1B01G403300.1	No hit		
TraesCS1B01G406000.1	1e‐26	YP_874698.1	Ribosomal protein S15 (chloroplast)
TraesCS1B01G407300.1	3e‐27	XP_020166758.1	GDSL esterase/lipase At5g45910‐like
TraesCS1B01G413800.1	1e‐53	KAE8794788.1	Putative sodium/metabolite cotransporter BASS1, chloroplastic
TraesCS1B01G417500.1	5e‐39	VAH22694.1	Unnamed protein product
TraesCS1B01G421200.1	4e‐34	AKJ77990.1	Endosperm transfer cell specific PR60 precursor
TraesCS1B01G423900.1	8e‐30	VAH22812.1	Unnamed protein product
TraesCS1B01G439200.1	2e‐38	XP_020170913.1	Disease resistance protein RPP13‐like
TraesCS1B01G451600.1	3e‐43	XP_020178665.1	Putative receptor‐like protein kinase At4g00960
TraesCS1B01G472200.1	No hit		
TraesCS1B01G473100.1	8e‐22	VAH23875.1	Unnamed protein product
TraesCS1B01G481000.1	2e‐44	VAH23967.1	Unnamed protein product
TraesCS1D01G379300.1	No hit		
TraesCS1D01G398900.1	9e‐28	XP_020178387.1	Tropinone reductase homolog At5g06060‐like

Note

The closest meaningful blast hit in GenBank nr is reported, if there is one. Otherwise the closest blast hit is reported, or no hit is reported if there was none at 1e‐20.

The third set of simulated reads was drawn from version 2.0 of the genome of the wheat‐pathogenic, ascomycete fungus *Zymoseptoria tritici* (Goodwin et al., 2011), which was downloaded from the Joint Genome Institute of the U.S. Department of Energy (https://mycocosm.jgi.doe.gov/Mycgr3/Mycgr3.home.html). Simulated long reads were produced in the same way as for wheat and assembled with unicycler. Ten homologous protein query sequences were downloaded from the GenBank nr database (Clark et al., 2016) and are listed in Table 2. These simulations demonstrated the behaviour of slag with a simple, relatively nonrepetitive genome. Assembly accuracy for the second and third simulated sets was measured by percentage identities in blastn output from contigs against the relevant reference genome.

**TABLE 2:** Target protein accessions from GenBank nr for local unicycler assemblies of simulated long reads derived from *Zymoseptoria tritici*

Accession	Description
AAD23831.1	NAD‐dependent formate dehydrogenase
AAD40111.1	3‐Isopropylmalate dehydrogenase
AAL30834.1	Anaphase‐promoting complex protein
ABD92790.2	Mitogen‐activated protein kinase
ABD94604.1	Nonribosomal peptide synthetase
ACS91347.1	Serine/threonine‐protein kinase
ADU79051.1	DNA lyase
AKA94181.1	Lanosterol 14‐alpha‐demethylase
ALP48286.1	RNA polymerase II second largest subunit
ANQ91929.1	Eburicol 14 alpha‐demethylase

### 2.3 Real data

Illumina reads (2 × 150 bp), PacBio long reads, a finished assembly and coordinates of gene models of *Zea mays* inbred line B73 were downloaded respectively from NCBI SRA and RefSeq as accessions ERR3288215–ERR3288217, ERR3288290–ERR3288295 and GCF_000005005.2, and the file GCF_000005005.2_B73_RefGen_v4_genomic.gff. Adapters and low‐quality reads were removed with fastp (Chen et al., 2018). The remaining 87.4 Gb of bases constituted 36.4× coverage of the 2.4‐Gb maize genome (Dong et al., 2017). Illumina reads (2 × 250 bp) and an assembly were downloaded from NCBI SRA and RefSeq for *Triticum aestivum* cv. “Stanley” as accessions SRR9125476 and GCA_903994154.1. Adapters and low‐quality regions were removed with htstream (Hunter et al., 2020), and leftover singleton reads were removed with a perl script. The remaining 178.5 Gb constituted 10.5× coverage of the 17‐Gb wheat genome (Montenegro et al., 2017). Fifty maize protein sequences were downloaded from the NCBI nr database for 10 enzyme activities, which are listed in Table 3. A local assembly was produced for each enzyme activity.

**TABLE 3:** Ten groups of maize enzyme accessions used to target local assemblies in maize and wheat

Activity	GenBank accession nos.
Cellulose synthase	NP_001104955.2, NP_001104956.2, NP_001104959.2, NP_001105236.2, NP_001105574.1, NP_001105672.1, NP_001292792.1
Ferredoxin	NP_001104851.1, NP_001136908.1, NP_001150750.1, NP_001336742.1, XP_020394593.1, XP_020405634.1
Hexokinase	NP_001123599.1, XP_008672065.1, XP_008674565.1, XP_008675068.1
Histone deacetylase	NP_001104901.1, NP_001105402.2, XP_008673398.1, XP_008677775.1, XP_020396306.1
Isocitrate dehydrogenase	AQK53344.1, AQK89292.1, AQK97039.1, AQK88693.1, NP_001295424.1, ONM16007.1, ONM58401.1
Peptidylprolylisomerase	AQK62104.1, AQK70996.1, AQL06400.1, ONM03151.1, ONM04876.1, ONM54033.1
Phosphoglucoisomerase	NP_001105368.1, XP_008651420.1
Phosphoglucomutase	NP_001105405.1, NP_001105703.1, XP_008675355.1, XP_020395615.1
Sucrose synthase	XP_008645119.1, XP_008679107.1, XP_020399433.1, XP_023156234.1
Transaminase	NP_001149818.2, NP_001278682.1, XP_008645517.1, XP_008668890.1, XP_008672129.1

### 2.4 Benchmarking vs. atram2 and srassembler

slag was benchmarked against atram2 version 2.1.1 and sraassembler version 1.0.0; attempts to run kollector failed due to unmet dependencies. Local assemblies were produced for the 10 enzyme activities listed in Table 3, using the real reads of maize “B73” and wheat “Stanley” described in Section 2.3, and also half and one‐quarter of the Stanley set. Read databases were prepared beforehand with makeblastdb, atram_preprocessor.py, or an invocation of srassembler itself, and read preparation was not included in runtime statistics. Each run was set up to execute 21 cycles of read retrieval and assembly. Under GNU/Linux and SLURM, each run was given exclusive access to a node of the Brown supercomputing cluster at Purdue University, and each was allowed to use 10 of the 24 cores on the node. Each run had use of the full 96 Gb of memory and all disk space accessible to the node for a maximum of 30 hr. However, the benchmarking jobs ran consecutively on different but identical nodes as assigned by the cluster's SLURM scheduler. Runtime and memory usage were reported with the SLURM sacct utility, and contig counts and lengths were noted from the assemblers’ output. Percentage identity with the “B73” and “Chinese Spring” (version 2.0) genomes was parsed from blastn output of contigs from the cycle that had produced the longest mean contig length.

slag used spades (Bankevich et al., 2012) to assemble “B73” reads and cap3 (Huang & Madan, 1999) to assemble “Stanley” reads. spades ran with phredoffset = 33 but otherwise default options. On the other hand, cap3 ran with options “‐b 20 ‐m 2 ‐n ‐4 ‐g 5 ‐s 600 ‐p 83 ‐o 40 ‐y 150 ‐z 3 ‐h 25 ‐j 70,” which tend to produce longer contigs at the risk of merging similar sequences. atram2 ran spades with atram2’s default options. Both slag and atram2 used a blast e‐value of 1e‐10 for initial alignments of the founding protein sequences to reads, but slag tightened the e‐value to 1e‐20 for subsequent alignments. slag used the “increment” option (extincrement = 10) to slowly increase the set of available reads at successive cycles, while atram2 was not similarly restricted.

In contrast to atram2, which had been installed previously on the Brown cluster, srassembler was compiled as srassembler_mpi for multithreaded execution using “make mpi with‐boost = [path to boost/1.64.0_gcc‐4.8.5/include].” Our attempts to run the alternative, supplied singularity mpi container failed to run multithreadedly. Also, initial trials revealed that the default configuration for srassembler did not work for large genomes, since far too many reads were retrieved from repetitive sequence to allow more than one cycle of retrieval and assembly. Instead, the benchmarking runs used “‐Z 400 ‐A 0 ‐n 21 ‐b 1 ‐x 0 ‐z 1 ‐d 500 ‐i 1000 ‐m 1000 ‐M 200000” and changes of vmatch parameters *e* and *l* to 1 and 45 for vmatch_protein_init, 1 and 100 for vmatch_extend_contig, 2 and 100 for vmatch_protein_vs_contigs, and 1 and 100 for vmatch_reads_vs_contigs. A single mismatch in a 100‐base match corresponded to a blastn e‐value of 5e‐51 in 20 test cases. These settings slowly increased the number of returned reads in consecutive cycles in accordance with slag’s strategy. Even with these settings, it was feasible to run srassembler only four times on maize reads, with a time limit of 80 hr per run. For the fourth srassembler run, which was seeded with isocitrate dehydrogenases, it was necessary to reduce parameters *i* and *m* to 700, since all first‐cycle contigs were shorter than 1000 bases.

For comparison of contig counts, the seeding protein sequences were also aligned to the B73 version 4.0 and Chinese Spring version 2.0 genomes with tblastx. A perl script then sorted the hit starts and ends in the genome scaffolds and called hits wherever a start was greater than the previous end. The script called genes wherever the gap between successive hits exceeded 10,000 bases.

### 2.5 Local assembly and bench verification of wheat sequences

Pyrosequencing (454) single reads of Chinese Spring wheat were downloaded from cerealsdb (Wilkinson et al., 2012). Four local assemblies were seeded with four Hessian fly‐responsive wheat sequences that encode dirigent‐like proteins. These included *HfrDrd* (Hessian fly‐responsive disease resistance dirigent‐like protein; GenBank accession JX501668), a nearly full‐length cDNA cloned from H9‐Iris wheat (Subramanyam et al., 2013); two related wheat dirigent sequences amplified using forward and reverse primers (Subramanyam et al., 2013) designed from *HfrDrd*, designated as *HfrDrd2* (GenBank accession KU178997.1) and *HfrDrd3* (KU170958.1); and 1.2 kb of upstream promoter sequence cloned from *HfrDrd2*. The 30 resulting contigs were mutually aligned with blastn (Zhang et al., 2000) at an e‐value of 1e‐08, and a custom Perl script found the depth of nonself blast hits of the contigs relative to one another at each nucleotide position and also the first and last positions where mutual alignment exceeded a minimum depth. The subsequence between these positions was scanned for single nucleotide polymorphism (SNP) positions with no nonself coverage. Wherever such positions were separated by at least a minimum product length, but less than a maximum product length, primer3 (Untergasser et al., 2012) attempted to find PCR primers such that the SNP was at or next to the 3′ end of one primer. The resulting contig‐specific tiling primers (Table 4 in part) for two *HfrDrd* and two *HfrDrdA* contigs were used to verify sequence assembly with staggered 600–1100‐bp amplicons.

**TABLE 4:** Primers for PCR validation of local assemblies

Target	Forward	Reverse
Contig 28‐1	CGCTTGCGTCTGTACTGTGTT	CGAAAGAACTCACGAAACACG
Contig 28‐2	GCTAACTTGCACTTGTTCTCG	CATATGATAAAACCCACCTCG
Contig 28‐3	CAAATGCATTAAATAGCGTGC	GGTCCTTGATGCTTGTGTTCT
Contig 16‐1	AGCTGAATGATAAATGCGGTA	TGGTGAGGTAGCAGGAACTACT
Contig 16‐2	TGCACTCCATTGATATTTCTCG	CCAAACCCAAAAGGAAAAGTC
Contig 21‐1	TGCTAAGTGCGTACAAAAGGAA	AATTGGTGCAAGAACAAGTGAC
Contig 21‐2	TTGCTATTTCTAGCCCCATCC	CTTGTGAAGCGTACACGAATG
Contig 24‐1	CTCGGAAGTTTATGGTAACCG	CCACCACTCAAACAACCACTA
Contig 24‐2	ATCCTTGGGTCAGGTTCTCAT	ACTTGAAGAAGCGTCAGCTCT
Contig 24‐3	TTTGCCTGTTGAGATGCATAG	ACGGTTGTACTTCCTCCATCA
Contig 24‐4	CCACTAGCGCAAATCCCTGTA	ACTGAAGGCAAGATGGGGTCT
Contig 24‐5	CTCGGTATTTTCTTGGGATTTG	CTTTGACTGGCGGTATACGAG
Contig 24‐6	CGGAGCTGTACAAGGAGAGAC	AGTGTCTATCCCGAAAGCAGA
Hfr‐1 genomic	ACACGCACACACACAATCCT	CAACACCCAGGCACGTACTA
Hfr‐1 promoter^a^	TGGTGGTCTCCAAGGTGAAAGACTGA	TTAGCTAGGATTGTGTGTGTGCGTGTGT
Hfr‐1 CS copy1	TCCAGAAAACCCCAGATGCT	CAACACCCAGGCACGTACTA
Hfr‐2 promoter	ACTGGCCTTCATGGCTGCCCAGATCCAA	CTCTCCTCGCTCCCTGCTTGCACGCTAC
CA666657 #1	CCTCTCCCGAACAATGGAAGGATTGC	GGCACGGATCTTGATGCAGAATGGAT
CA666657 #2	AAGGTTCATCAAAATCAATTTCGTTGTCG	CGGAGGATGGGATGCTCTCAATGACAA

Note

Forward and reverse PCR primers are listed 5′–3′.

Abbreviation: CS, Chinese Spring.

^a^ Nested Genomewalker primers are listed.

For PCR, each 50‐µl reaction contained 200 µm dNTPs (Bioline USA), 190 ng of genomic DNA template extracted from the wheat line “Chinese Spring,” 0.5 µm of forward and reverse primers, 1× Phusion HF buffer, and 1 U of Phusion High Fidelity DNA polymerase (New England Biolabs). PCR was performed with the following parameters: 98°C denaturation for 30 s; 30 cycles of 98°C for 30 s, contig‐specific annealing temperature (3°C above the lower melting temperature of the two primers [*T*_m_] as determined by the *T*_m_ calculator at www.neb.com) for 30 s, 72°C for 30 s; followed by 10 min of extension at 72°C. The PCR amplicons were run on 1% agarose gels in Tris‐acetate‐EDTA buffer and stained with ethidium bromide to test for a single PCR product for each reaction. The PCR amplicons were purified using the MinElute PCR Purification kit (Qiagen) and then Sanger sequenced directly by the Purdue Genomics Core Facility.

Local assemblies from Chinese Spring reads (Wilkinson et al., 2012) were also seeded with an expressed sequence tag (EST) of unknown function (GenBank accession CA666657) and with two unrelated Hessian fly‐responsive genes, *Hfr*‐*1* (AF483596.1; Williams et al., 2002) and *Hfr*‐*2* (AY587018.1; Puthoff et al., 2005). The contigs most similar to the seeding sequences were identified with blastn. Contig indels and SNPs were identified with multalin (Corpet, 1988) relative to the seeding sequences.

Genomic, homologous and upstream promoter sequences of *Hfr*‐*1* were confirmed by cloning. The genomic sequence of *Hfr*‐*1* was amplified from 100 ng of H9‐Iris wheat genomic DNA and its homologs from 100 ng of Chinese Spring wheat genomic DNA by PCR using Ex Taq polymerase (Takara Bio) following the manufacturer's instructions. Primers (Table 4) for PCR were designed from the nearly full‐length cDNA sequence (GenBank accession AF483596). PCR was carried out with the following parameters: 95°C denaturation for 1 min; 35 cycles of 95°C for 1 min, 55°C for 1 min, 72°C for 1 min; followed by 7 min extension at 72°C. The promoter region upstream of *Hfr*‐*1* was cloned from 100 ng of H9‐Iris wheat genomic DNA using the Genome Walker Kit (Clontech) following the manufacturer's instructions. To verify the *Hfr*‐*2* promoter sequence generated by the local assembly program, PCR primers were designed from the assembly‐generated sequence to amplify the promoter region, with the forward primer 1.5 kb upstream of the 5′ untranslated region (UTR), and the reverse primer within the *Hfr*‐*2* coding region (Table 4). PCR was carried out using 100 ng of H9‐Iris wheat genomic DNA with PfuTurbo hot‐start DNA polymerase (Agilent Technologies) following the manufacturer's instructions. PCR was performed with the following parameters: 95°C denaturation for 2 min; 30 cycles of 95°C for 30 s, 60°C for 30 s, 72°C for 1 min 45 s; followed by 10 min extension at 72°C. The 1.7‐kb PCR amplicon was gel purified with the QIAquick gel extraction kit (Qiagen). For the EST (GenBank accession CA666657), two sets of PCR primers were designed (Table 4) from the most‐matching contig sequence generated by the local assembly program to amplify a longer sequence from H9‐Iris wheat. The first set of PCR primers was designed such that the amplicon would overlap the original seed sequence by 145 bp and advance 1.6 kb in the 3′ direction and the second set of PCR primers was designed to overlap the first amplicon by 151 bp and proceed 3′ an additional 1.6 kb, including 750 bp of the section matching known genes by blastn search. PCR was performed as it was for *Hfr*‐*2*. All amplicons were cloned into pCR4 TOPO TA vector (Invitrogen) and sequenced by the Purdue Genomics Core Facility.

## 3 RESULTS

### 3.1 Choice of read selection and stopping criterion

Initial trials showed that contigs could grow, shrink and grow again over consecutive cycles, and therefore it was not a good idea to stop slag once a target‐matching contig had stopped growing. Therefore, trials were run for a set number of cycles. The choice of 21 cycles seemed long enough to exhaust growth of target‐matching contigs in most instances, so all further trials were run with 21 cycles. Furthermore, the fixed‐increment read‐selection criterion produced the longest contigs in early trials, and an appropriate choice of increment increased the chance that a maximum‐length target‐matching contig would be reported before shrinkage began. Therefore, all further trials used the fixed‐increment option.

### 3.2 Contig growth

**FIGURE 1:**
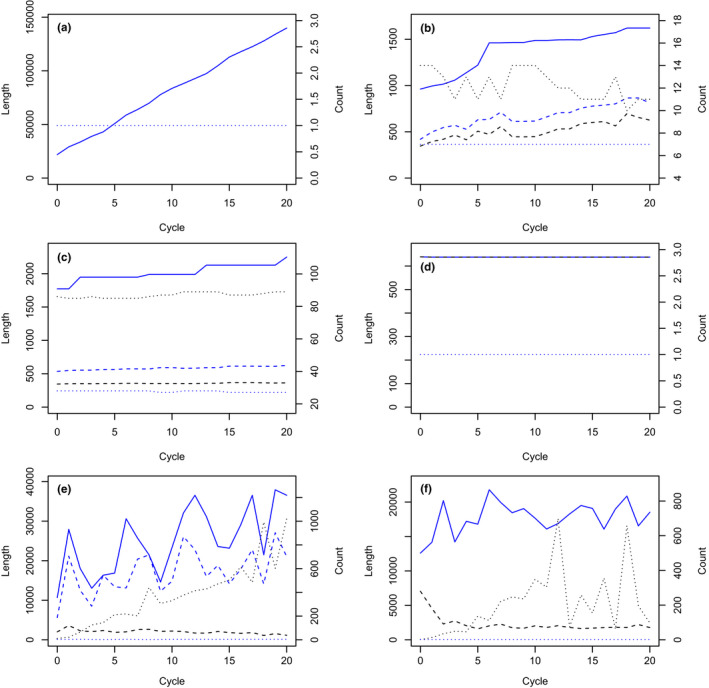
Examples of changes in contig length and count over cycles of read retrieval and assembly. Solid, dashed and dotted blue lines are respectively the longest length, mean length and count of target‐matching contigs. Solid, dashed and dotted black lines are respectively the longest length, mean length and count for all generated contigs. (a) Monotonically increasing contig length with cycle number in *Zymoseptoria tritici*. Simulated long reads were assembled with unicycler. A single contig was produced at each cycle. (b) Relatively monotonic contig growth in wheat for simulated short reads assembled with spades. (c) Stepwise contig growth of maize transaminase for actual short reads assembled with cap3. (d) Constant contig size for singly fragmented, simulated long reads in wheat assembled with phrap. (e) Irregular, generally increasing contig size for singly fragmented, simulated long reads in wheat assembled with phrap. (f) Irregular, generally level contig size for singly fragmented simulated long reads in wheat assembled with phrap

**FIGURE 2:**
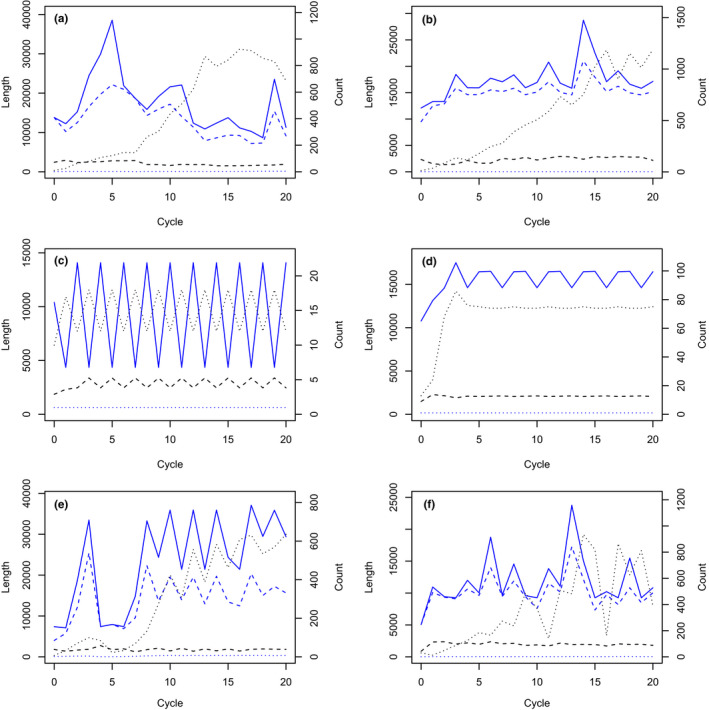
More examples of changes in contig length and count over cycles of read retrieval and assembly. Line types and colours conform to Figure 1. (a) Irregular contig size, generally decreasing between cycles 5 and 18, for singly fragmented simulated long reads in wheat assembled with phrap. (b) Conspicuously peaked contig length at cycle 14 for singly fragmented simulated long reads in wheat assembled with phrap. (c) Alternating contig lengths for singly fragmented simulated long reads in wheat assembled with phrap. (d) Limit cycle with three values of contig length for singly fragmented simulated long reads in wheat assembled with phrap. (e) Brief alternation of contig lengths for cycles 10–14 from singly fragmented simulated long reads in wheat assembled with phrap. (f) A second instance of greatly fluctuating length and count of contigs similar to Figure 1f

The naively expected behaviour is a monotonic increase in contig length over cycles, at least for target‐matching contigs. Such a monotonic increase was observed only for unicycler assembly of long reads from the small, relatively nonrepetitive genome of *Zymoseptoria tritici* (Figure 1a). In all other instances, the competitive recruitment of reads to target‐matching contigs and nonmatching contigs affected and ultimately curtailed the growth of target‐matching contigs. Relatively monotonic growth (Figure 1b) could grade through episodic, stepwise growth (Figure 1c) to no growth at all when the first cycle exhausted all overlapping, matching reads (Figure 1d). More generally, contigs shrank after one or more cycles as additional reads fed the growth of nonmatching contigs that recruited reads away from matching contigs, or as a long contig was split in the subsequent cycle. The resulting trend could be upward (Figure 1e), level (Figure 1f) or downward (Figure 2a). Single‐cycle peaks (Figure 2b) and limit cycles (Figure 2c,d) occurred occasionally. Sometimes growth could break free of a limit cycle as a higher fraction of reads was assembled (Figure 2e).

### 3.3 On‐target percentage

The fraction of contigs that matched target sequence varied widely among target sequences, as can be seen from the counts of target‐matching and overall contigs in Figures 1 and 2. Generally the number of target‐matching contigs was nearly constant while nontarget‐matching contigs increased in later cycles, as is particularly evident in Figure 2b, e. Consecutive cycles could differ greatly in total contig count as contigs were merged, split or assembled as distinct (Figures 1f and 2f).

### 3.4 Contig length

In most cases the longest contig was the same for target‐matching and overall contigs (Table 5). Where they differed, the longest overall generally came from a run‐ending cycle that failed to produce a target‐matching contig. Lengths varied 1000‐fold among assemblers and read types. Longer reads resulted in longer contigs, and modern assemblers produced longer contigs than cap3, while requiring greater read depth to produce any assembly at all. In the particular case of fragmented long reads assembled with phrap, double fragmentation yielded shorter contigs than single fragmentation because the “minprogress” variable had been set, which ended the run once consecutive cycles produced longest matching contigs fewer than 100 nucleotides different in length.

**TABLE 5:** Means over all target sequences for identities and maximum contig lengths obtained from any cycle of local assembly

Species	Assembler	ReadL	ReadC	Comp	NMat	MatContLen	NAll	AllContLen	Identities
*T. aestivum*	spades	2 × 150 bp	60×	49/50	3.66	1619.10 ± 28.68	6.18	1619.10 ± 28.68	98.76
*T. aestivum*	cap3	2 × 150 bp	5×	50/50	2.48	1112.18 ± 57.38	7.53	1113.52 ± 57.13	97.30
*T. aestivum*	phrap	2 × 150 bp	5×	50/50	3.82	946.16 ± 29.53	7.30	946.16 ± 29.53	98.56
*T. aestivum*	cap3	600 bp	20×	50/50	2.66	4681.46 ± 213.62	3.81	4681.46 ± 213.62	99.43
*T. aestivum*	phrap	600 bp	20×	50/50	2.77	4901.34 ± 188.09	4.35	4901.34 ± 188.09	99.77
*T. aestivum*	canu	7–14 kb	60×	6/50	1.66	27,554.75 ± 6007.7	2.97	27,607.17 ± 6021.7	98.69
*T. aestivum*	unicycler	7–14 kb	60×	17/50	1.21	115,521.1 ± 6132.5	1.22	115,521.1 ± 6132.5	90.05
*T. aestivum*	unicycler	7–14 kb	100×	21/50	1.26	31,326.21 ± 4345.4	1.36	31,554.13 ± 4330.4	90.00
*T. aestivum*	cap3	7–14 kbf1A	5×	30/50	4.12	1425.65 ± 71.89	68.62	1818.12 ± 68.06	90.20
*T. aestivum*	phrap	7–14 kbf1	5×	47/50	1.89	27,053.51 ± 1521.38	313.30	28,880.51 ± 1351.12	95.42
*T. aestivum*	cap3	7–14 kbf2	5×	49/50	8.37	7396.69 ± 266.05	454.75	9026.37 ± 295.26	90.07
*T. aestivum*	phrap	7–14 kbf2	5×	49/50	8.15	9920.18 ± 451.68	361.15	14,756.82 ± 804.90	90.00
*T. aestivum*	spades,P	2 × 150 bp	60×	19/22	3.55	1662.05 ± 149.41	5.71	1662.05 ± 149.41	98.29
*Z. tritici*	unicycler,P	7–14 kb	60×	10/10	1.01	139,855.4 ± 3057.9	1.02	139,855.4 ± 3057.9	90.80
*Z. mays*	cap3,P	7–85 kbf1	35×	9/10	12.38	2054.60 ± 245.53	734.14	3065.60 ± 274.16	90.93
*Z. mays*	phrap,p	7–85 kbf1	35×	6/10	5.86	69,011.70 ± 4275.95	1576.62	72,174.80 ± 4206.15	95.39
*Z. mays*	canu,P	7–85 kb	35×	1/10	3.20	63,517.33 ± 5922.12	5.08	63,731.67 ± 5807.39	99.54
*Z. mays*	spades,P	2 × 150 bp	32×	10/10	29.15	5260.60 ± 368.88	70.06	5260.60 ± 368.88	99.57
*Z. mays*	cap3,P	2 × 150 bp	7×	10/10	37.87	2602.00 ± 354.17	96.52	2602.00 ± 354.17	98.92
*Z. mays*	phrap,p	2 × 150 bp	7×	10/10	14.34	3294.10 ± 442.11	54.20	3294.10 ± 442.11	97.11

Note

Key to column headings and codes: Species, *Triticum aestivum*, *Zea mays* and *Zymoseptoria tritici*; Assembler, self‐explanatory; ReadL, read length; ReadC, read coverage; Comp, number of founding query accessions that completed all 21 cycles; NMat, mean count of contigs that matched the founding query sequence per cycle over all completed cycles; MatContLen, mean length of longest contigs that matched the founding query sequence, ±*SEM*; NAll, mean count of all contigs per cycle over all completed cycles; AllContLen, mean length of all longest contigs, ±*SEM*, taken from the file with the longest contig that matched the founding query sequence; Identities, percentage identity of founding query sequence to the closest‐matching contig. Means were taken over all founding query accessions that completed 21 cycles. Symbol P in the Assembler column designates a protein query. Symbol f1 in the read‐length column designates single fragmentation of long reads to 600‐base fragments, while f2 designates double fragmentation of long reads to 490‐ and 610‐base fragments that were gathered together to create the illusion of doubled read coverage.

Percentage identities and maximum lengths of target‐matching maize contigs varied by enzyme group (Table 6), to display the variation in number of given target sequences and the size of the underlying gene families, of which cellulose synthase is the largest (Little et al., 2018; Penning et al., 2019). Contig length varies by assembler much as in Table 5, and for any given assembler, contig length varies among the enzyme groups. spades produced longer contigs than phrap or cap3 on short reads, possibly because it could use paired‐end relationships that were necessarily broken to run phrap or cap3. phrap easily produced much longer assemblies on long reads, where physical linkage in the long reads reduced the number of similar reads derived from unlinked loci that were summoned to the subsequent cycle of assembly. There was enough consistency in the ranking of short‐read contig lengths for a given assembler to suggest that the length of unique sequence varied around the different loci.

**TABLE 6:** Length and percentage nucleotide identity to the B73 genome assembly for longest contigs produced at any cycle of local assembly

Group	*N*	SRcap3	SRphrap	SRspades	LR1cap3	LR1phrap	LRcanu
Cellulose synthase	7	4341	5983	6934	2435	80,550	37,544
		0.987	0.959	0.998	0.913	0.955	0.994
Ferredoxin	6	1899	2534	3917	1685	72,297	67,820
		0.995	0.984	0.998	0.899	0.916	0.998
Hexokinase	4	1817	2040	6488	1425	51,448	73,014
		0.987	0.970	0.996	0.912	0.961	0.998
Histone deacetylase	5	1950	2325	4574	3652	94,221	37,815
		0.993	0.977	0.996	0.904	0.963	0.993
Isocitrate dehydrogenase	7	1670	2070	6430	2107	58,619	68,339
		0.988	0.984	0.995	0.908	0.963	0.997
Peptidylprolylisomerase	6	1919	2179	4493	2157	70,564	85,620
		0.987	0.957	0.993	0.915	0.955	0.999
Phosphoglucoisomerase	2	2029	4313	5932	1108	78,023	—^a^
		0.974	0.960	0.997	0.916	0.952	—
Phosphoglucomutase	4	4627	4567	4851	1656	52,436	85,954
		0.997	0.993	0.994	0.904	0.955	0.996
sucrose synthase	4	3519	4417	5471	2904	72,015	57,554
		0.998	0.977	0.995	0.908	0.941	0.993
Transaminase	5	2249	2513	3516	1417	59,944	57,996
		0.992	0.985	0.993	0.911	0.958	0.995
Matching contigs Nonmatching contigs	50	373	140	285	129	85	26
		2	1	3	0	0	0

Note

Key to fields: Group, enzyme activity or count of contigs that did or did not match the genome of B73; *N*, count of target protein sequences; SRcap3, cap3 assembly of separated 2 × 150‐bp reads; SRphrap, phrap assembly of separated 2 × 150‐bp reads; SRspades, spades assembly of 2 × 150‐bp reads; LR1cap3, cap3 assembly of PacBio reads singly fragmented to 600‐bp chunks; LR1phrap, phrap assembly of PacBio reads singly fragmented to 600‐bp chunks; LRcanu, canu assembly of intact PacBio reads. Read coverage and mean contig accuracy are given in Table 5. Key to rows: for each enzyme function, the top row is the length of the longest contig obtained with the field‐designated assembler, and the bottom row is the fraction of bases that match the B73 genome over all target‐matching contigs. The nonmatching contigs match bacterial variants of isocitrate dehydrogenase and phosphoglucomutase.

^a^ canu assembly failed for phosphoglucoisomerase.

For comparison, the median length of a high‐confidence wheat gene model, as calculated from the gff3 file for the IWGSC genome assembly (International Wheat Genome Sequencing Consortium, 2018), is 1922 bp, and the median length of the 50 targeted gene models is 2203.5 bp. The longest local wheat contigs based on short reads were generally shorter than the median size of a wheat gene. Similarly, the median length of a maize gene model in the B73 genome assembly (Jiao et al., 2017) is 2402 bp, and the median length for the targeted gene models was 3598 bp. The median of longest cap3 local maize assemblies based on short reads (1989.5 bp) was shorter than either of these, as were seven of 10 longest contigs based on short reads.

The ratios of longest contig lengths to first‐cycle contig lengths for seven combinations of read length and assembler are given in Table 7. These ratios are binned in increments of 0.4. Few initial contigs were more than doubled in length at their longest, except with phrap and once‐fragmented long reads. There was no evident reason for phrap not similarly lengthening the doubly‐fragmented reads.

**TABLE 7:** Distribution of lengthening of longest contigs for 50 seeding sequences over 21 cycles of slag operating on simulated reads from *Triticum aestivum*

Program	Read length	Read depth	1.00–1.39	1.40–1.79	1.80–2.19	2.20–2.59	2.60–2.99	3.00+
cap3	2 × 150	5×	7	22	10	5	2	4
phrap	2 × 150	5×	9	16	12	6	3	4
spades	2 × 150	60×	0	32	10	1	2	4
cap3	7–14kf	5×	29	4	1	0	0	0
phrap	7–14 kf	5×	3	6	6	9	5	18
cap3	7–14kd	5×	20	10	8	7	4	0
phrap	7–14kd	5×	42	5	2	0	0	0

Note

The six numerical columns at right are counts of seeding sequences that produced a ratio of maximum contig length to initial contig length within the stated range. Code kf indicates single fragmentation, while kd indicates double fragmentation.

### 3.5 Contig accuracy

Contig accuracy, measured as percentage matching to reference genomes, is reported in the last column of Table 5 and the lower row of each couplet in Table 6. As expected, increased read accuracy and depth of coverage both favoured accurate assembly. Assemblers that identify and attempt to correct erroneous reads (i.e., canu and spades) outperformed unicycler, which pastes pieces of reads together and then tries to polish the result (Wick et al., 2017). Neither phrap nor cap3 was consistently as accurate as canu or spades, and neither was consistently more accurate than the other with short reads; phrap was more accurate with low‐coverage short reads in wheat and less so in maize. However, phrap appeared to be markedly more accurate than cap3 for once‐fragmented long reads in both wheat and maize, where cap3 was only as accurate as the reads themselves. Neither phrap nor cap3 improved upon the accuracy of doubly fragmented long reads. There is no obvious explanation for the relatively lower accuracy of ferredoxin with singly fragmented long reads (Table 6).

The cap3, phrap and spades assemblies were less accurate than the individual short reads in their input for wheat, and only spades matched the accuracy expected of individual short reads in maize. For short reads from maize, the mean percentage identity of retrieved reads for the cycles that produced the longest target‐matching contigs was 99.43% against the B73 reference genome, vs. 99.57%, 98.92% and 97.11%, respectively, for the spades, cap3 and phrap assemblies collectively based on the same reads. For short reads from wheat, the mean percentage identity of retrieved reads was 99.38% against the Chinese Spring reference genome, vs, 98.76%, 97.30% and 98.56% respectively for the spades, cap3 and phrap assemblies collectively based on the same reads.

Initial inspection suggested that the error frequency was higher near the ends of contigs. Therefore, mismatches of contig and reference sequence were tabulated in the closest blastn alignment over three intervals: the terminal 100 bases at each end of the contig and the remaining bases covering the middle of the contig. Over all contigs produced with cap3 from short reads, the mean mismatch frequency was 1.35% per base in the terminal segments and 0.60% per base in the middle segment. This difference was significant by a paired‐data *t* test (*p* = 2.3e‐10, *df* = 372).

Assembly accuracy might differ between contig subsequences that match a gene and subsequences from the same contig that match outside a gene. To test this possibility, we extracted the entire sequence of the gene models in the B73 reference genome that matched the short‐read cap3 contigs, aligned the contigs to the gene models with blastn, and compared the output percentage identities with the percentage identities from contigs aligned to the whole reference genome, using only the closest blast hit for each contig. Either both identities were equal, as expected for contigs that matched entirely within a gene model, or the identities were unequal, as expected if the contig extended beyond the gene. The percentage identities were compared by a two‐tailed, paired‐data *t* test in R for all contigs and also for only contigs whose identities differed. For all target groups, mean contig identity to the whole genome (99.13%) exceeded mean identity to gene models only (98.72%, *p* = 4e‐05, *df* = 364), while for contigs with unequal identities, mean identity to the whole genome (98.44%) more obviously exceeded mean identity to gene models only (95.66%, *p* = 1e‐05, *df* = 53). For only the very large family of cellulose synthases, mean identity to the whole genome (99.04%) and to gene models only (98.73%, *p* = 0.028, *df* = 133) were almost identical to the values for all groups.

**FIGURE 3:**
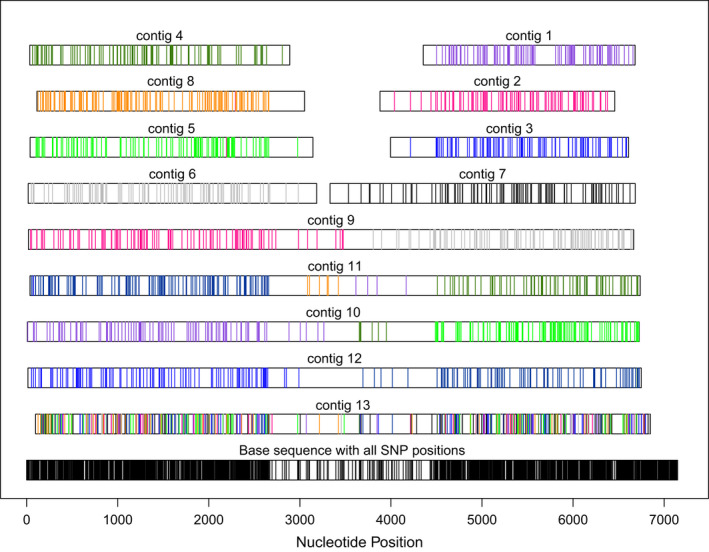
Detailed alignment of a phrap assembly of simulated pyrosequencing reads to the simulated genome from which the reads were sampled. Thirteen contigs represent nine simulated alleles of a 7‐kb locus. Polymorphic nucleotide (SNP) positions are colour‐coded by allele of origin. The bottom contig in black shows superimposed all variant positions, and a central, conserved region is evident by a paucity of SNPs, as intended. This central region exceeds any read in length, it is under‐represented in the assembly, and the assembled copies of it in contigs 10 and 11 are consensuses of two different alleles. Contig 13 is a consensus sequence of multiple alleles throughout. Contigs 1–8 represent a single left or right flank of an allele, and contigs 9–12 are flanks of different alleles joined in the central region. The depicted contig depth (9) equals the number of alleles (9), which was typical for alleles that differed this much in sequence

The simulations with a 7‐kb random founding sequence reveal the most frequent types of misassembly that can occur with phrap and possibly other assemblers. Figure 3 is illustrative. In this example, medium‐length reads (300–900 bp) were simulated from nine variant alleles of the nine‐fold duplicated locus, which contained an 1800‐base relatively conserved core subsequence and more divergent flanking subsequences as detailed in the Materials and Methods. The figure appears as horizontal bars that represent the contigs aligned relative to a common ancestor (base sequence) at the bottom. SNP positions are indicated by vertical line segments on each bar. The central region is clearly evident by its lower SNP frequency. SNP positions on the contig bars are colour coded by their source allele. A completely accurate reconstruction of the alleles would require nine contiguous horizontal bars, each with a uniform colour of SNP positions. In Figure 3, none of the bars appears this way. Instead, there are 13 contigs, where eight contigs represent individual flanking regions with an almost entirely uniform colouring of SNP positions, and four contigs are chimeras of flanking regions from different alleles. Contig 13 differs; it is a consensus of all nine alleles and thus does not reconstruct any single allele. Such consensus contigs were uncommon among independent simulations, but association of flanking regions in chimeric contigs was seemingly random, as expected since no read spanned the central conserved region.

In an example with phrap assembly of real pyrosequencing reads of Chinese Spring wheat, two instances of probable misassembly involved an inverted‐repeat structure. The local assembly was seeded with GenBank protein accession BAC99512.1, a putative caffeic acid 3‐*O*‐methyltransferase from japonica rice. The affected contigs were 3090 and 6327 nucleotides long, and in each contig the repeats themselves were closely similar but not identical. The inverted repeats flanked a short, core sequence of 56 bases in the first contig and 37 bases in the second contig. The first 20 bases of both core sequences were identical. Three reads fully spanned the core bases in the first contig, and one read fully spanned the core bases in the second, although 169 and 162 additional reads respectively matched the core bases in part at an e‐value of 0.01. None of the structure matched any entry for miniature inverted‐repeat transposable elements in P‐MITE (Chen et al., 2014).

### 3.6 Contig polishing

**FIGURE 4:**
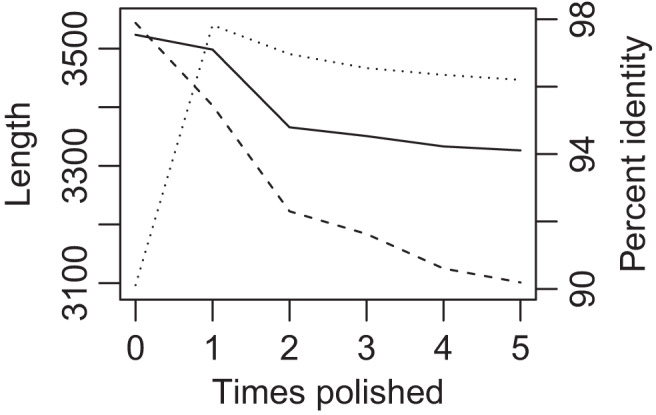
Effect of short‐read polishing on contig length and accuracy in singly‐fragmented long reads of wheat assembled with cap3. Solid line is contig length, dashed line is blast‐hit length, and dotted line is percentage identity vs. the Chinese Spring genome. Means are presented for all target‐matching contigs from all 21 cycles of assembly

An initial test, which involved four seed sequences, considered the optimal number of iterations to polish long‐read‐based contigs with much more accurate short reads. At each cycle, the contigs that otherwise would have advanced directly to the next cycle were subjected to five iterations of racon (Vaser et al., 2017). As shown for three target sequences in Figure 4, the first iteration of polishing improved accuracy from 90% to 97%, but each subsequent iteration slightly reduced accuracy to only 96% after five rounds. Each round of polishing slightly decreased mean contig length and mean length of blastn alignment. Furthermore, five rounds of polishing took about 10 times more runtime than the rest of slag.

### 3.7 Comparison to atram2 and srassembler

slag was compared under standard conditions with atram2 for the 10 maize enzyme activities given in Table 3 and the four read data sets described in Section 2.4. Because of its slowness, srassembler was compared using four of the 10 proteins, ferredoxins, hexokinases, histone deacetylases and isocitrate dehydrogenases. Means of several contig and runtime descriptors are given in Table 8. The first row gives the number of runs that completed at least one cycle of read retrieval and assembly. slag completed 21 cycles for all proteins and data sets, whereas atram2 failed to produce an assembly with one enzyme group with half‐Stanley and nine enzyme groups with quarter‐Stanley. The only group that yielded any atram2 contigs with quarter‐Stanley was cellulose synthases, the largest known gene family in grasses, where paralogues increased the depth of highly similar reads. Thus slag with cap3 was more robust to low read coverage than atram2 with spades. srassembler yielded B73 contigs with all four tested enzyme groups, but isocitrate dehydrogenases required a parameter change (*i* and *m* to 700 from 1000) to work.

**TABLE 8:** Comparative contig and runtime statistics for slag, atram2 and srassembler

Property	SLAGB73	aTRAMB73	SRAB73	SLAGfStanley	aTRAMfStanley	SLAGhStanley	aTRAMhStanley	SLAGqStanley	aTRAMqStanley
Enzymes completed	10	10	4	10	10	10	9	10	1
Contig count	25.50	57.3	4.00	44.00	101.9	36.30	48.33	28.70	131.00
Contig/loci ratio	0.73	0.96	0.46	1.34	1.99	1.10	0.94	0.87	2.55
Mean length	2043.21	1826.45	1862.10	1084.02	1295.19	1130.38	987.71	1335.64	1067.95
Maximum length	5260.60	5252.20	2858.25	3434.70	3552.0	3125.50	2758.11	3178.80	4622.00
Percentage matched	99.65	99.47	100.00	97.86	99.16	97.83	98.57	97.50	98.41
Number of cycles	21.00	15.9	6.00	21.00	11.50	21.00	4.89	21.00	1.00
Seconds per cycle	2406.02	1469.00	32271.97	677.34	590.77	166.87	1930.72	83.88	962.00
Resident memory (bytes)	1.386e + 10	8.707e + 09	1.199e + 07	2.520e + 10	3.578e + 09	1.275e + 10	2.280e + 10	6.382e + 09	2.720e + 09
Virtual memory (bytes)	5.813e + 10	1.846e + 11	5.834e + 08	7.314e + 11	9.964e + 09	2.983e + 10	7.253e + 10	7.210e + 10	8.776e + 09
Maximum page faults	29.50	151.70	0.00	30.70	214.67	26.40	130.67	30.60	271.00

Note

Column headings B73, fStanley, hStanley and qStanley refer respectively to the read sets from maize “B73” and the full, half and quarter read sets from wheat “Stanley.” The enzymes are given in Table 3. All rows below the first are means over the number of runs given in the first row. Percentage match refers to contig identity with the B73 or Chinese Spring reference genomes. Memory is the maximum at any point during the run.

In Table 8, the number of atram contigs was filtered for matching the seeding protein sequence at 1e‐10, while the counted slag contigs matched the seeding sequence at 1e‐20. The calculation of the contig/locus ratio is given in Table 9. There was no consistent relationship of contig count and estimated locus count over the four data sets, although atram deviated more highly. As expected from the stringent matching required to recruit reads, srassembler produced fewer contigs than slag or atram2, and the fraction of blastable loci returned as contigs was also lower. srassembler was bound by the number of contigs that passed the minimum length criterion in the initial cycle, and only once did the number at later cycles exceed that.

**TABLE 9:** Calculation of expected loci and contig/loci ratio

Program	slag	atram	sra	slag	atram	slag	atram	slag	atram
Read set	B73	B73	B73	CS full	CS full	CS half	CS half	CS quarter	CS quarter
e‐value	1e‐20	1e‐10	1e‐50	1e‐20	1e‐10	1e‐20	1e‐10	1e‐20	1e‐10
Contig count	25.50	57.3	4.00	44.00	101.9	36.30	48.33	28.70	131.00
Loci in genome	34.9	59.4	8.75	32.9	51.3	32.9	51.3	32.9	51.3
Contig:loci ratio	0.73	0.96	0.46	1.34	1.99	1.10	0.94	0.87	2.55

Note

Contig count came from Table 8. Number of loci was estimated from the distribution of blastn hits in the appropriate genome, with a minimum of 10,000 bases between loci.

slag was slower than atram2 on average, while both slag and atram2 were much faster than srassembler, which was hindered by the slowness of vmatch alignment. The mean speed advantage of atram2 conceals its highly variable mean runtime per cycle (Table 10), which ranged from 103 to 2584 s for “B73” and 205 to 7966 s for the full “Stanley” read set. slag was faster than atram2 for four of 10 enzymes in “B73,” four of 10 enzymes in full “Stanley” and all nine enzymes in half “Stanley.” In the last case, atram2 spent most of its time preparing and running the protein‐to‐nucleotide alignment at the first cycle, and subsequent cycles were quick. It appears from Table 10 that slag runtime scaled at least superlinearly with size of the reads database. In the extreme cases, slag was 16.8 times slower than atram2 in “B73” transaminases to 17.5 times faster in half‐”Stanley” sucrose synthases. There was no significant Pearson correlation of atram runtime to slag runtime for the “B73” read set (*r* = −.298, *p* = .40), the full “Stanley” read set (*r* = −.207, *p* = .57) or the half‐”Stanley” read set (*r* = .480, *p* = .19).

**TABLE 10:** Mean cycle durations for slag and atram2 subdivided by enzyme and read set

Read set	Enzyme	slag cycle duration (s)	atram2 cycle duration (s)	Ratio
Zea	Cellulose synthase	3589.90	855.33	4.197
Zea	Ferredoxin	1609.24	3049.90	0.528
Zea	Hexokinase	1720.48	2023.14	0.850
Zea	Histone deacetylase	2494.33	2583.86	0.965
Zea	Isocitrate dehydrogenase	1827.24	344.33	5.307
Zea	Peptidylprolyl isomerase	4696.67	496.86	9.453
Zea	Phosphoglucoisomerase	1658.00	103.11	16.080
Zea	Phosphoglucomutase	1750.10	2292.52	0.763
Zea	Sucrose synthase	2657.57	1082.00	2.456
Zea	Transaminase	2055.24	122.00	16.846
Full Stanley	Cellulose synthase	1312.67	252.43	5.200
Full Stanley	Ferredoxin	223.05	793.50	0.281
Full Stanley	Hexokinase	414.52	308.25	1.345
Full Stanley	Histone deacetylase	389.29	230.57	1.688
Full Stanley	Isocitrate dehydrogenase	978.90	460.17	2.127
Full Stanley	Peptidylprolyl isomerase	582.81	205.48	2.836
Full Stanley	Phosphoglucoisomerase	346.67	1512.31	0.229
Full Stanley	Phosphoglucomutase	215.52	1836.75	0.117
Full Stanley	Sucrose synthase	1777.62	400.89	4.434
Full Stanley	Transaminase	531.05	7966.31	0.067
hemiStanley	Cellulose synthase	407.29	1458.00	0.279
hemiStanley	Ferredoxin	80.62	155.00	0.520
hemiStanley	Hexokinase	164.71	509.00	0.324
hemiStanley	Histone deacetylase	90.81	669.00	0.136
hemiStanley	Isocitrate dehydrogenase	196.10	638.00	0.307
hemiStanley	Peptidylprolyl isomerase	155.67	—	—
hemiStanley	Phosphoglucoisomerase	104.33	143.44	0.727
hemiStanley	Phosphoglucomutase	71.62	220.00	0.326
hemiStanley	Sucrose synthase	217.33	3820.62	0.057
hemiStanley	Transaminase	178.95	667.00	0.268

Note

Duration included the initial alignment of protein queries to the nucleotide reads database.

Further examination revealed that only 10 of 40 atram2 runs completed all 21 cycles (Table 11). Four more runs stopped when consecutive cycles did not alter the contigs, which is a normal stopping criterion for atram2. The remaining 26 runs failed in some way, mostly during spades assembly, where 10 runs did not yield contigs at all and 10 more failed in subsequent cycles, which prematurely limited contig length. In six cycles, the database was locked; all six involved the largest, full‐”Stanley” read set. It is not obvious if this happened because this was the largest read set, or because all these runs happened consecutively over a single time period. The case of exceeded time limit, full‐Stanley transaminases, completed 13 cycles and had collected 2 million reads in the 14th cycle when the 30 hr expired. However, no contigs matched the seeding protein sequences after the fifth cycle. Instead, the assembly had gone off into repetitive sequence.

**TABLE 11:** Ending status of atram2 runs

Outcome	B73	Stanley		
	B73	Full Stanley	Half‐Stanley	Quarter‐Stanley
21 completed	6	2	1	0
No contigs updated	3	1	0	0
Assembly failed after first cycle	1	0	8	1
Assembly failed at first cycle	0	0	1	9
Database locked	0	6	0	0
Out of time	0	1	0	0

Note

The wall time limit for each run was 30 hr. atram2 stopped in four instances where contigs did not grow between cycles of read retrieval. In all but one instance of assembly failure, spades’s exit status was 21, which was apparently related to insufficient read depth for the diversity of reads.

slag’s memory usage was proportional to the size of the reads database (Table 8). slag used somewhat more memory than atram2 except with half‐”Stanley.” Thus atram2’s memory usage was not proportional to the size of the read set, and actually was greater for half‐”Stanley” than for full‐”Stanley.” In contrast, srassembler used less than 0.1% as much memory as slag. Neither slag nor atram2 consistently used more virtual memory than the other, but virtual memory requires disk access, which greatly slows program execution. Variation in virtual memory usage might account for most of the variation in atram2’s run time. srassembler used less than 1% as much virtual memory as slag. Page faults reflect the need to read in pages of virtual memory and thus the ability of a program to cache needed information. Here, atram2 had five to nine times the transfer of pages to and from disk that slag had, and srassembler appeared not to page much at all.

### 3.8 Laboratory verification of assemblies in wheat

Local assemblies were verified in the laboratory for four unrelated wheat genes: Hessian fly‐responsive disease resistance dirigent‐like protein (*HrfDrd*), Hessian fly responsive gene 1 (*Hfr*‐*1*), Hessian fly responsive gene 2 (*Hfr*‐*2*) and an anonymous EST (GenBank accession CA666657). Four *HfrDrd* variants (*HfrDrd*, *HfrDrd2*, *HfrDrd3* and *HfrDrd2*‐promoter) seeded local assembly, and they collectively yielded 30 contigs. Based on blastn alignments, eight contigs most closely matched *HfrDrd*, 16 contigs most closely matched *HfrDrd2*, none most closely matched *HfrDrd3*, one contig most closely matched the *HfrDrd2*‐promoter and five contigs did not match any of the seed sequences at an e‐value of 1e‐05 or closer.

Contig‐specific primers for *HfrDrd2* contig28 amplified three overlapping fragments representing 2228 bp of sequence, including the entire coding region. The sequencing results shared 99.9% identity with the locally assembled sequence. Two overlapping fragments were amplified from *HfrDrd2* contig 16 representing 1823 bp of sequence, 241 bp of which included the first exon of *HfrDrd2*, while the remaining sequence was 5′ to the coding region. The sequencing result was 99.9% identical to the locally assembled sequence. Two overlapping fragments were amplified from *HfrDrd* contig 21 representing 757 bp of sequence, which spanned a region 5′ to the coding region through part of exon 2. The resulting sequence was 92.6% identical to the assembled sequence. Finally, six tiled fragments were amplified from *HfrDrd* contig 24 representing 3999 bp of sequence, all 3′ to the coding region and 97.9% identical to the locally assembled sequence.

Local assembly seeded with the 1267‐bp coding region of *Hfr*‐*1*, previously cloned from wheat line H9‐Iris, provided eight contigs from 490 to 7667 nucleotides in length that shared 85%–100% identity with the seeding sequence. The perfectly matching contig also matched 1050 bp of upstream promoter sequence of *Hfr*‐*1*, which had been obtained previously from H9‐Iris by genome walking, and it provided an additional 2548 bp of 5′ sequence. Apart from one SNP, the three locally assembled introns matched the introns that had been previously cloned from H9‐Iris. Two previously cloned copies of *Hfr*‐*1* from Chinese Spring wheat were also identified among the eight locally assembled contigs. One copy matched the same contig as the H9‐Iris version at 99.9% (one single‐base indel in 1254 bp total), and the other copy matched a different contig at 99.7% (four SNPs in 1252 bp total).

To obtain the promoter sequence of *Hfr*‐*2*, local assembly was seeded with 1727 bp of *Hfr*‐*2* coding sequence. Eight of the resulting 16 contigs were 99% identical to the seed sequence. Primers were designed from the two contigs that went furthest 5′ to the seeding sequence. These two contigs differed by a 2‐bp indel within the 5′ UTR and a 16‐bp indel ~130 bp 5′ to the 5′ UTR. The obtained clones included both versions, confirming both sequences and providing promoters for two copies of the gene.

Local assembly produced three contigs that were 93%, 95% and 99% identical to the 392‐bp EST seeding sequence. The 99%‐matching contig was 4.5 kb in length and matched *Aegilops tauschii* cytosolic acetyl‐CoA carboxylase (*Acc*‐*2*) and putative amino acid permease towards the 3′ end (bases 3421–4284; e‐value = 0.0 by blastn). The cloned regions differed from the local assembly by only two single‐base indels, confirming that the annotated section and EST belong to the same gene.

## 4 DISCUSSION

slag robustly produced local assemblies with at least one assembler in each of the tested situations, including challenging examples from hexaploid wheat and simulations with long‐read sequencing depth as low as 5×. The usefulness of the assemblies depends upon the intended purpose, read accuracy, sequencing depth and the choice of assembler parameters, but in favourable instances the assemblies suffice for primer design and estimation of variant count in multigene families.

In general, contigs can stop growing because of gaps in read coverage resulting from low read depth or removal of particular reads as putative contaminants based on GC content. In unique sequence, contigs can grow continuously to chromosomal length. However, in repeat‐rich sequence, contig growth is self‐limiting and depends on the relative length of reads and repeats. Nascent contigs compete for reads, and contigs in repetitive regions tend to be a consensus of multiple loci, so that they do not match or join the ends of contigs centred in unique regions.

Contig accuracy with slag depends upon read accuracy, read depth and assembly method. An assembler can be confused by many similar but differing variant reads from paralogues, especially if the assembler attempts to correct read errors on the basis of variant frequency. This might explain why cap3 assemblies with ~97% accuracy were less accurate than the individual Illumina reads that were assembled.

Two plausible uses of slag are (i) identification of genes in a novel genome and (ii) obtaining sequence as a basis of targeted genetic markers for linkage or deletion mapping or analysis of population structure. Either use can require PCR primers or baiting sequences for the production of targeted, accurate reads. Primers require a more accurate assembly than baits. If *x* is the probability that an individual base was correctly called in the assembly, then the probability of two exactly matching primers of lengths *m* and *n* is *x* ^(^ *^m^* ^+^ *^n^* ^)^. For two 20‐base primers and *x* = 0.990, this is only 0.669 and increases to 0.818 for *x* = 0.995. When highly accurate reads are available, it might be better to derive primers from individual reads that match desired sites in the assembled contig. Alternatively, target baits with >85% sequence identity probably suffice if given lowered hybridization temperature, according to the hybridization study of He et al. (2005).

The intended use determines the assembly strategy to use. Exon‐specific baits or primers do not require abutting repetitive elements, and relatively short, coding‐sequence contigs are not only sufficient but desirable. Complete genes or promoters need longer contigs, even at the risk of a significant frequency of chimeric misassemblies of similar loci, since comparison to the source reads is always possible. Calling multiple alleles in a heterozygous polyploid requires high accuracy and very probably long reads to get sufficient polymorphism without the chimerism exhibited in Figure 3.

Local assemblers can follow two distinct strategies to include additional reads in a growing contig. One strategy, used in an earlier version of slag, uses some portion of each end of a contig to query the reads database, and then assembles the end‐matching reads with the contig to produce the next iteration of the contig. However, if the assembly only uses the querying end of the contig, with the expectation of joining it later to the core of the contig, there is a risk of losing homology to the seeding sequence. This conceivably happened with atram2 with full‐”Stanley” transaminases. The other strategy, used currently in slag, is to query reads with the whole contig and then assemble only the matching reads. The latter strategy allows contigs to grow and shrink in successive iterations as the number of reads increases in increments.

A fair comparison of slag to other local assemblers is difficult, because slag and atram2 have many parameters that potentially interact to affect the rate of contig growth and the propagation of nontarget contigs. In the tests reported here, slag’s increment parameter was probably set too conservatively to match the rate of contig growth often seen with atram2. slag’s increment setting ideally should increase with greater read depth, and in the benchmarking tests it was constant across read depths. Furthermore, setting the cap3 parameter –*p* to the default value of 0.93 would have increased contig count and accuracy at the expense of contig length. For assembling distinct alleles from a heterozygote, setting –*p* to 0.97 would be necessary.

The database sharding strategy of atram2 promises efficient search of read sets, and atram2 was much faster than slag when that strategy worked as intended. However, there were instances where the reverse was true, where atram2 became bogged down in its management of virtual memory or started to assemble contigs based on repetitive sequence, as evidenced by the presence of contigs that did not match the seeding protein sequences. slag was more robust than atram2: slag completed all 21 prescribed cycles for all tested enzymes and read sets, while atram2 completed only 13 of 40 runs without errors, and only nine of those went the full 21 cycles. Admittedly, the benchmarking tests emphasized the ability to assemble shallow read depths, but even with deep read coverage, atram2 sometimes failed because of database locking.

Comparison to srassembler was greatly impeded by srassembler’s slowness in selecting reads with vmatch, despite its splitting the reads database about as much as atram2. srassembler seemed to produce very good assemblies as far as they went, but it was impractical to test it thoroughly.

## AUTHOR CONTRIBUTION

C.F.C. designed, wrote and tested slag. J.A.N. and S.S. performed the laboratory confirmation of the *Hfr* and *HfrDrd* sequences. C.F.C., S.S. and J.A.N. wrote the manuscript with editorial help from S.B.G. S.B.G. and C.E.W. supported the project.

## CONFLICT OF INTEREST

The authors declare that there is no conflict of interest. Mention of trademarks or vendors does not constitute an endorsement by the U.S. Federal Government; other products or vendors may be equally suitable.

## Data Availability

Configuration files and results of slag runs have been deposited at datadryad.org with identifier https://doi.org/10.5061/dryad.0p2ngf22s. The Sanger sequences for *Hfr*‐*1*, *HfrDrd*‐*2* promoter, *HfrDrd*‐*2* and *HfrDrd*‐*3*, have been deposited in NCBI GenBank as accessions KU214205.1, KU178997.1, KU170957.1 and KU170958.1, respectively. slag is available as source code and a Singularity container at https://github.com/cfcrane/SLAG.
